# Estrogen mediates inflammatory role of mast cells in endometriosis pathophysiology

**DOI:** 10.3389/fimmu.2022.961599

**Published:** 2022-08-09

**Authors:** Alison McCallion, Yasmin Nasirzadeh, Harshavardhan Lingegowda, Jessica E. Miller, Kasra Khalaj, SooHyun Ahn, Stephany P. Monsanto, Mallikarjun Bidarimath, Danielle J. Sisnett, Andrew W. Craig, Steven L. Young, Bruce A. Lessey, Madhuri Koti, Chandrakant Tayade

**Affiliations:** ^1^ Department of Biomedical and Molecular Sciences, Queen’s University, Kingston, ON, Canada; ^2^ Department of Obstetrics and Gynecology, University of North Carolina, Chapel Hill, NC, Canada; ^3^ Department of Obstetrics and Gynecology, Wake Forest Baptist Health, Winston-Salem, NC, United States

**Keywords:** endometriosis, immune microenvironment, mast cell recruitment and maturation, stem cell factor, immune crosstalk, estrogenic inflammation

## Abstract

Endometriosis is an estrogen dependent, chronic inflammatory disease characterized by the growth of endometrial lining outside of the uterus. Mast cells have emerged as key players in regulating not only allergic responses but also other mechanisms such as angiogenesis, fibrosis, and pain. The influence of estrogen on mast cell function has also been recognized as a potential factor driving disease pathophysiology in number of allergic and chronic inflammatory conditions. However, precise information is lacking on the cross talk between endocrine and immune factors within the endometriotic lesions and whether that contributes to the involvement of mast cells with disease pathophysiology. In this study, we observed a significant increase in mast cell numbers within endometriotic lesions compared to matched eutopic endometrium from the same patients. Compared to eutopic endometrium, endometriotic lesions had significantly higher levels of stem cell factor (SCF), a potent growth factor critical for mast cell expansion, differentiation, and survival for tissue resident mast cells. Targeted mRNA Q-PCR array revealed that the endometriotic lesions harbour microenvironment (upregulation of CPA3, VCAM1, CCL2, CMA1, CCR1, and KITLG) that is conducive to mast cells recruitment and subsequent differentiation. To examine cross-talk of mast cells within the endometriotic lesion microenvironment, endometriotic epithelial cells (12Z) and endometrial stromal cells (hESC) incubated with mast cell-conditioned media showed significantly increased production of pro-inflammatory and chemokinetic cytokines. To further understand the impact of estrogen on mast cells in endometriosis, we induced endometriosis in C57BL/6 mice. Mature mast cells were significantly higher in peritoneal fluid of estrogen-treated mice compared to untreated mice within the sham operated groups. Mouse endometriotic lesion tissue revealed several genes (qRT-PCR) relevant in mast cell biology significantly upregulated in the estrogen treated, endometriosis-induced group compared to control endometrium. The endometriotic lesions from estrogen treated mice also had significantly higher density of Alcian blue stained mast cells compared to untreated lesions or control endometrium. Collectively, these findings suggest that endometriotic lesions provide a microenvironment necessary for recruitment and differentiation of mast cells. In turn, mast cells potentially release pro-inflammatory mediators that contribute to chronic pelvic pain and endometriosis disease progression.

## Introduction

Endometriosis (EMS) is an estrogen (E2) dependent, multifaceted, chronic inflammatory gynecologic disease affecting 7-10% of women of reproductive age. It is characterized by the presence of endometriotic tissue at ectopic locations; most commonly the peritoneal wall, ovaries, bladder, or colon (1). The symptomatology of endometriosis is heterogenous, but mainly includes pelvic pain and infertility

The lack of diagnostic and therapeutic options is partially owing to a poor understanding of EMS pathophysiology, which is multifaceted and largely elusive, though immune dysregulation is considered one of the major predisposing factors. Multiple immune cells and their products have been implicated, including mast cells (MCs) (2), in the pathophysiology of EMS. Mast cells are tissue resident immune cells that do not mature until reaching their target tissue and are dependent on stem cell factor (SCF) to develop, migrate to target tissue, and to survive/proliferate (3). MCs have been implicated in the cell proliferation, angiogenesis, and fibrosis of EMS lesions, as well as pain symptoms (4–9). In fact, multiple studies have acknowledged the high prevalence of allergies, asthma, and other MC-related immune disorders in EMS patients (10–12), and elevated levels of both SCF and mast cell tryptase have been reported in peritoneal fluid of women with EMS compared to healthy controls (13, 14).

The estrogen dominant nature of EMS impacts immune functioning as estrogen and progesterone have numerous immunomodulatory effects, though the immune-endocrine relationship within EMS has yet to be fully defined. Interestingly, endometriotic lesions have been shown to produce high levels of E2 (15), and there is evidence that MCs mature and degranulate upon stimulation with E2 (16). However, a knowledge gap remains as to whether MCs are recruited and differentiated by specific chemokines and growth factors from the lesion microenvironment, or if they are simply present as bystanders.

In this study, we analyzed eutopic endometrium and ectopic lesions from endometriosis patients to quantify the number of mast cells, concentration of SCF, and transcript levels of genes relevant to mast cell biology. We also investigated the cell signalling cross-talk of human mast cells and endometriotic epithelial and endometrial stromal cells *in vitro* under hormonal conditions. In addition, we used a syngeneic mouse model of endometriosis to investigate mast cell frequency in the murine peritoneal immune cell population and lesion tissue after endometriosis induction with or without estrogen treatment. Our findings suggest potential recruitment of mast cells to the lesions and contribution from mast cells to the development of lesions through their support of angiogenesis and fibrosis, and point to agonistic effects of estrogen in the pathophysiological role of mast cells in endometriosis.

## Materials and methods

### Human samples

Human eutopic endometrium, endometriosis tissue, and peritoneal fluid were obtained from endometriosis patients (n=14, stage III-IV) who underwent laparoscopic excision surgery after informed consent at the Greenville Health System, Greenville, SC, USA. Endometrial samples from healthy women were obtained (n=8) as controls from University of North Carolina (Chapel Hill, NC, USA). The endometrial samples, both from patients and healthy women matched for secretory stage of the menstrual cycle were obtained by Pipelle sampling, as per standard procedure. All women were free from hormonal therapy for 3 months prior to the collection of samples. Study is approved by the Queen’s University Health Sciences Research Ethics Board.

### Analysis of expression of selected mast cell-related genes in human patient samples and mouse endometriotic lesions

To investigate expression of genes relevant to mast cell recruitment, differentiation, and activation between endometriosis patients and healthy controls, we customized a qPCR panel using custom RT^2^ profiler PCR array (Qiagen, Canada). Eleven genes of interest (IL1B, VCAM1, TAC1, TPSAB1, KITLG, CPA3, IL3, CCL2, CMA1, TNF, CCR1) and five housekeeping genes/controls (ACTB, GAPDH, human genomic DNA contamination control, reverse transcription control, positive PCR control) were selected for this panel (**Table 1**). RNA was extracted from endometriotic lesions (n=9), matched eutopic endometrium (n=8), and normal healthy endometrium (n=10) tissue lysates using the Norgen total RNA purification kit (Norgen Biotek, Canada #37500). Total RNA of all samples were stored at -80˚C until reverse transcribed into cDNA. Equal amounts of RNA (500 ng) were used to synthesize cDNA using RT2 first strand synthesis kit (#330404, Qiagen, Canada). We performed qPCR following manufacturers’ protocol using RT^2^ SYBR green master mix and equal amounts of cDNA for each sample (#330503, Qiagen).

**Table 1:** Genes targeted in custom RT^2^ Profiler PCR array.

Gene	Protein encoded	Function in mast cell biology	EMS lesion vs. normal	P value	EMS lesion vs. EMS eutopic	P value	EMS eutopic vs. normal	P value
IL1B	Interleukin 1-β	Recruitment	+	0.427	*+*	1.7E-5	+	0.162
IL3	Interleukin 3	Recruitment, differentiation	+	0.103	*+*	5E-10	+	0.44
VCAM1	Vascular adhesion protein 1	Recruitment	*+*	2.0E-6	/	0.5	+	0.533
CCL2	C-C Motif chemokine ligand 2	Recruitment	*+*	2.06E-4	*+*	4.97E-5	+	0.358
TAC1	Preprotachykinin-1 AKA “Substance P”	Differentiation	+	0.173	*+*	0.0049	+	0.253
CMA1	Mast cell chymase 1	Differentiation	*+*	0.0073	*+*	2.3E-8	+	0.0627
TPSAB1	Mast cell tryptase	Differentiation, activation	*+*	0.0053	*+*	1.86E-11	+	0.126
TNF	Tumor necrosis factor	Activation/function	*+*	0.0204	*+*	1.41E-7	+	0.452
KITLG	Stem cell factor (kit ligand; c-kit)	Differentiation, recruitment	*+*	0.0047	+	0.27	+	0.969
CCR1	C-C Motif chemokine receptor 1	Activation/function	*+*	*0.0014*	*+*	*1.0E-15*	*+*	*0.0369*
CPA3	Mast cell carboxypeptidase 3	Activation/function	*+*	*1.32E-4*	*+*	0.0525	+	0.251
GAPDH	Glyceraldehyde 3-phosphate dehydrogenase	Housekeeping gene	n/a	n/a	n/a	n/a	n/a	n/a
ACTB	β-actin	Housekeeping gene	n/a	n/a	n/a	n/a	n/a	n/a

“**+**”, significantly upregulated (fold change > 1.0; p<0.05), “+”, upregulated (fold change higher than 1.0), “-”, downregulated (fold change lower than -1.0), “/”, no observed difference (-1.0 < fold change < 1.0).

A similar panel of gene targets was used to analyze endometriotic lesion tissues harvested from endometriosis-induced mice (IL1B, VCAM1, TAC1, TPSAB1, KITLG, CPA3, IL3, CCL2, CMA1, TNF, CCR1, FCERIa, ITGB7, ESR1, ESR2). The same protocol was applied with RT^2^ SYBR green master mix and equal amounts of cDNA for each sample (#330503, Qiagen). Using ACTB as a reference gene, fold change and relative transcript levels were determined by ΔΔCT analysis method performed in Microsoft Excel with p values determined by multiple unpaired t-tests with FDR (Q= 1.00%) multiple comparisons correction in GraphPad Prism^®^ (GraphPad Software Inc., CA, USA).

### Immunohistochemistry of human mast cell tryptase

Paraffin embedded human eutopic endometrium and paired ectopic endometriotic lesions were obtained from patients at Kingston General Hospital, Queen’s University (Kingston, ON, Canada). The blocks were prepared into slides at 5μm per section and were used for immunohistochemistry with monoclonal mouse anti-human mast cell tryptase antibody (Dako, Burlington, ON, Canada, n=6). The sections were subjected to antigen retrieval in sodium citrate buffer (pH 6.0) for 25 minutes at 95˚C. To prevent non-specific protein interaction, the sections were incubated with 1% BSA for 15 minutes, followed by 3% H_2_O_2_ treatment for 10 minutes to block endogenous peroxidase activity. Avidin block was added for 15 minutes followed by a Biotin block (Anti-mouse cell and tissue staining secondary kit, R&D Inc., Minneapolis, USA). Subsequently, monoclonal mouse anti-human mast cell tryptase primary antibody (Dako, Burlington, ON, Canada) was added at a concentration of 1:200 in PBS overnight in a humidified chamber at 4˚C. A biotinylated secondary antibody (R&D systems, Minneapolis, USA) was added for 30 minutes the following day. Following a PBS wash, HSS-conjugated HRP was added for 30 minutes. The sections were again washed and stained with DAB chromogen, and then counterstained with Harris’ Hematoxylin (Fisher Scientific, Ottawa, ON) for 10 seconds. The slides were coverslipped with permamount mounting medium. Tryptase positive mast cells were visualized on Zeiss Axio Observer microscope (Oberkochen, Germany) and counted with the aid of AxioVision system software. Mast cells were counted in four representative regions and averaged by tissue area per mm^2^ in human ectopic lesions and matched eutopic endometrium.

### Stem cell factor ELISA

Concentrations of SCF in matched eutopic and ectopic lesions tissue lysates, and peritoneal fluid from patients (n=14) were measured using Stem Cell Factor Human ELISA kit (Abcam, Cambridge, UK). 50μl of standard or sample (tissue lysate or peritoneal fluid) was added to each well and incubated for two hours at room temperature. Subsequently, 50μl of biotin antibody was added to each well and incubated for one hour. Additionally, 50μl of Streptavidin-Peroxidase Conjugate was added and incubated for 30 minutes at room temperature. Next, 50μl of the chromogen substrate was added to each well and incubated for 15 minutes or until the optimal blue color density developed. Addition of 50μl of stop solution to each well concluded color development and the color intensity was measured at 450 nm. Efficiency was determined by use of standard curve and linear regression analysis. The SCF sample concentration was determined using the standard curve, adjusted for the dilution factor of the original sample.

### Cytokine analysis for endometriotic epithelial cells (12Z) and human endometrial stromal cells cultured with human mast cell (HMC-1)-conditioned media

First 12Z cells (human endometriotic epithelial cells) (17) were cultured in DMEM-F12 (Gibco™, Cat. 11320022 + 10% FBS, 1% sodium pyruvate, 1% penicillin/streptomycin) with estrogen or progesterone in concentrations of 1x10^-6^ M, 1x10^-7^ M, or 1x10^-8^ M as well as a combination of both hormones at 1x10^-7^ M. As well, hESC cells were cultured in PriGrow IV growth medium (Applied Biological Materials Inc., Cat. TM004 + 10% charcoal stripped FBS, 1% L-Glutamine, 1% penicillin/streptomycin) and given the same hormone treatments as listed. Supernatant and cells were collected after 24 hours. Supernatant was analyzed using commercially available multiplex cytokine analysis panel that includes GM-CSF, IFN-γ, IL-1β, IL-1ra, IL-2, IL-4, IL-5, IL-6, IL-8, IL-10, IL-12(p40), IL-12(p70), IL-13, MCP-1, and TNF-α (Eve Technologies, Alberta, Canada, HD-15) to establish baseline production of inflammatory cytokines by these cells under hormonal conditions. HMC-1 cells (human mast cell leukemia cell line 1, kindly gifted by Dr. Joseph Butterfield, Mayo Clinic, MN) were cultured at 1x10^6^ cells/mL in IMDM (EMD Millipore Cat. SLM-06-B, 10% FBS, 1.2 mM alpha-thioglycerol, 1% penicillin/streptomycin) with hormone treatments of the same concentrations for 48 hours, then cells and supernatant were collected. Expression of sex hormone receptor genes (ESR1, ESR2, PR, AR) in HMC-1, 12Z and hESC cells was verified by RT-PCR analysis. An aliquot of supernatant from HMC-1 cells were analyzed with the HD-15 panel, and remainder of the supernatant was used as conditioned media for 12Z and hESC cells (media adjusted for each cell line’s growth medium needs). The 12Z and hESC cells were incubated for 24 hours and supernatant was collected for cytokine analysis with the HD-15 panel. One-way ANOVA statistical analysis was performed using GraphPad Prism^®^ (GraphPad Software Inc., CA, USA).

### Syngeneic mouse model of endometriosis

This experiment was done using our established syngeneic mouse model of endometriosis (18, 19). C57BL/6 mice (n=10) were acquired from The Jackson Laboratory, and housed in cages of two to three mice. Experiments were approved by the Queen’s Institutional Animal Care Committee. Donor mice were sacrificed, uterine horns removed, and placed in PBS. The endometrium was punched into 3mm^3^ segments using a dermal biopsy punch and kept on ice in PBS until surgically implanted in recipient mice. Prior to surgery, recipient mice were anesthetized in an isofluorane vaporizer. A small incision was made in the abdomen of each recipient mouse and two 3mm^3^ segments of donor mouse endometrium were grafted in the left side of the peritoneal cavity of recipient mice using 3M VetBond adhesive. Postoperative fluid therapy and analgesics were given, and lesions were allowed to establish for 14 days. Of the EMS-induced mice (n=10), five were treated with a 17-β-estradiol pellet (1.5mg, zero-order 21-day release, Cat. E-121, Innovative Research of America, Sarasota, FL, USA) implanted subcutaneously at the animal’s neck three days prior to EMS induction surgery. Sham surgeries were performed on groups with (n=5) and without (n=5) the estrogen treatment. Blood samples were collected on day 0, day 7, and at endpoint (day 14). Peritoneal lavage and endometriotic lesions were harvested at endpoint.

### Cytokine analysis in mouse blood plasma

Blood was collected from the submandibular vein of all experimental mice into K_2_EDTA-coated tubes (BD Biosciences, 365974) and immediately placed on ice. The blood was centrifuged for 15 minutes at 900g and 4°C, and plasma was collected. Aliquots of plasma were diluted twofold in PBS and stored at –80 °C. Blood plasma cytokines were analysed through a commercially available multiplex panel including Eotaxin, G-CSF, GM-CSF, IFN-γ, IL-1α, IL-1β, IL-2, IL-3, IL-4, IL-5, IL-6, IL-7, IL-9, IL-10, IL-12 (p40), IL-12 (p70), IL-13, IL-15, IL-17A, IP-10, CXCL1, LIF, LIX, MCP-1, M-CSF, MIG, MIP-1alpha, MIP-1beta, MIP-2, RANTES, TNF-α, and VEGF (Eve Technologies, Alberta, Canada, MD-31).

### Flow cytometry of mouse peritoneal immune cells

Peritoneal immune cells were collected by peritoneal lavage with 5 mL of PBS and immediately placed on ice. After red blood cell lysis (Thermo Fisher, A1049201), samples were passed through a 70-μm nylon cell strainer to create a single cell suspension (Corning, 10054-456). To assess cell number and viability, 10 µl aliquots of samples were stained trypan blue and analyzed by Countess™ II Automated Cell Counter (Applied Biosystems, Massachusetts, USA). For each sample, 5x10^5^ cells were resuspended in flow staining buffer (2% FBS in PBS). Cells were then stained with PE-labeled anti-mouse CD45 (30-F11 clone, Biolegend, 103105), FITC-labeled anti-mouse FCERIα (MAR-1 clone, Biolegend, 134305), Alexa700-labeled anti-mouse CD117 (2B8 clone, Biolegend, 105846), PE/Cy7-labeled anti-mouse integrin-β7 (FIB504 clone, Biolegend, 321241), allophycocyanin (APC)-labeled anti-mouse CD11b (M1/70 clone, Biolegend, 101212), and Zombie Aqua viability dye (Biolegend, 423101) for 30 minutes on ice. After staining, cells were fixed using a commercially available fixation buffer (eBioscience, 00-8222-49) as per manufacturer’s instructions. Samples were analyzed using CytoFLEX S (Beckman Coulter, USA). Isotype, fluorescent minus one (FMO), and negative controls were used to guide data analysis using the FlowJo v10 software (Ashland, Oregon, USA). Mature mast cells were identified as CD45^+^ FCERIα^+^ CD117^+^ CD11b^—^, and MCcp were identified as CD45^+^ CD117^+^ integrin- β7^+^.

### Histochemistry of mouse endometriotic lesions

Harvested ectopic lesions as well as donor uterine segments were fixed in 4% paraformaldehyde prior to tissue processing. Paraffin embedded ectopic lesions and control uterine fragments were sectioned (10 µm thickness) and deparaffinised using Citrisolv (Fisher Scientific, Massachusettes, USA) and decreasing concentration of ethanol. Sections were stained with Alcian blue solution (pH 2.5) for 30 minutes and counterstained using nuclear fast red solution for 5 minutes and coverslipped using a mounting medium. Analysis was performed with HALO AI Software (Indica Labs, Albuquerque, NM, USA) using an area quantification algorithm.

### Immunohistochemistry of mouse endometriotic lesions

Immunohistochemistry (CD31 and Ki67) for mouse lesions was performed using an automated slide stainer i (BenchMark XT, Ventana Medical System Inc, Tucson, AZ, USA). After antigen retrieval, sections were incubated with primary anti-CD31 (1:100, New England Biolabs, 77699S) or anti-Ki67 (1:1000, Abcam, ab15580) monoclonal antibodies. Sections then received suitable secondary antibody staining and Ultrablue DAB detection kit was used for color development (Ventana Medical System Inc). Sections were counterstained with nuclear stain (hematoxylin) and bluing reagent for 4 minutes before coverslip addition. Analysis was performed with HALO AI Software (Indica Labs, Albuquerque, NM, USA) using a cytonuclear analysis algorithm in the case of Ki67 and area quantification algorithm for CD31 stain.

### Statistical Analyses

Statistical analysis of mast cell quantification was performed using Graph Pad Prism^®^ (Graph Pad Software Inc., CA, USA). One-way ANOVA statistical analyses were performed using GraphPad Prism^®^ (GraphPad Software Inc., CA, USA) to compare multiple groups in histochemistry area quantification analysis, cytokine analysis, and in flow cytometry analysis. Data was refined with ROUT outlier test (Q= 10%) and statistical analyses were only performed on cleaned data. When applicable, unpaired student’s t-test with Welch’s correction or one-way ANOVA with Tukey’s multiple comparisons were used. Error bars represent the standard error of the mean (SEM). A p value of <0.05 was considered significant.

## Results

### Human endometriotic lesions express significantly upregulated mast cell-related genes compared to eutopic or normal healthy endometrium

**Figure 1:**
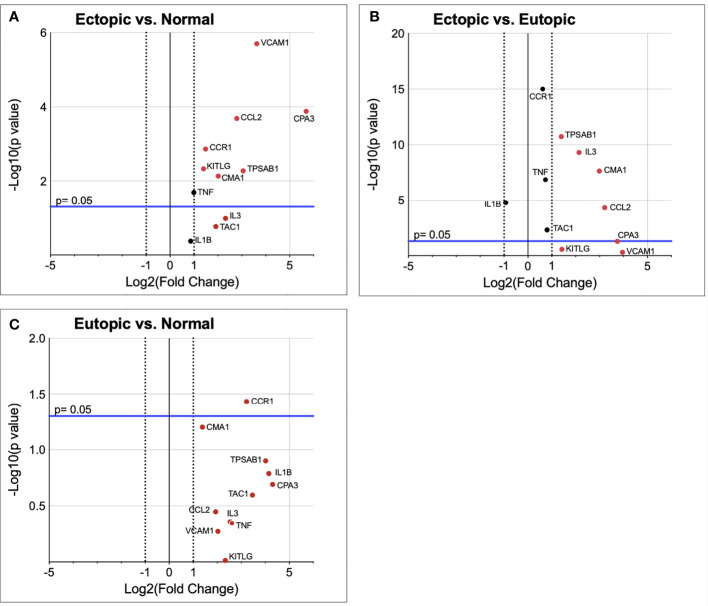
Mast cell relevant genes are upregulated in endometriotic lesion tissue. Gene transcript fold change in ectopic endometriotic lesion (n=9) vs normal healthy endometrium (n=10) **(A)**, eutopic endometrium (n=8) of endometriosis patients vs normal healthy endometrium **(B)**, and ectopic endometriotic lesion vs eutopic endometrium of endometriosis patients **(C)**. Vertical dotted lines represent a fold change of ±2, where data points outside this range have shown a fold change of more than 2.0. The blue horizontal line denotes p= 0.05 in -Log10, where data points above the blue line are significantly upregulated in the tested group.

To understand whether endometriotic lesions contain a microenvironment conducive to mast cell recruitment and differentiation, we performed targeted qPCR analysis examining the transcriptomic abundance of 11 genes relevant to mast cell biology (**Table 1**). Significant upregulation of TPSAB1, CPA3, VCAM1, CCL2, CMA1, CCR1, and KITLG (p<0.05), as well as TNF (p=0.06), was found in endometriotic lesions as compared to normal healthy endometrium (**Figure 1A**). Comparison of endometriotic lesions with matched eutopic endometrium from same patients revealed significant upregulation of TPSAB1, CMA1, IL3, and CCL2 (p<0.05, **Figure 1B**). Only CCR1was found to be significantly upregulated in patient eutopic endometrium compared to normal healthy endometrium (**Figure 1C**).

### Human endometriotic lesions have significantly higher numbers of activated mast cells

**Figure 2:**
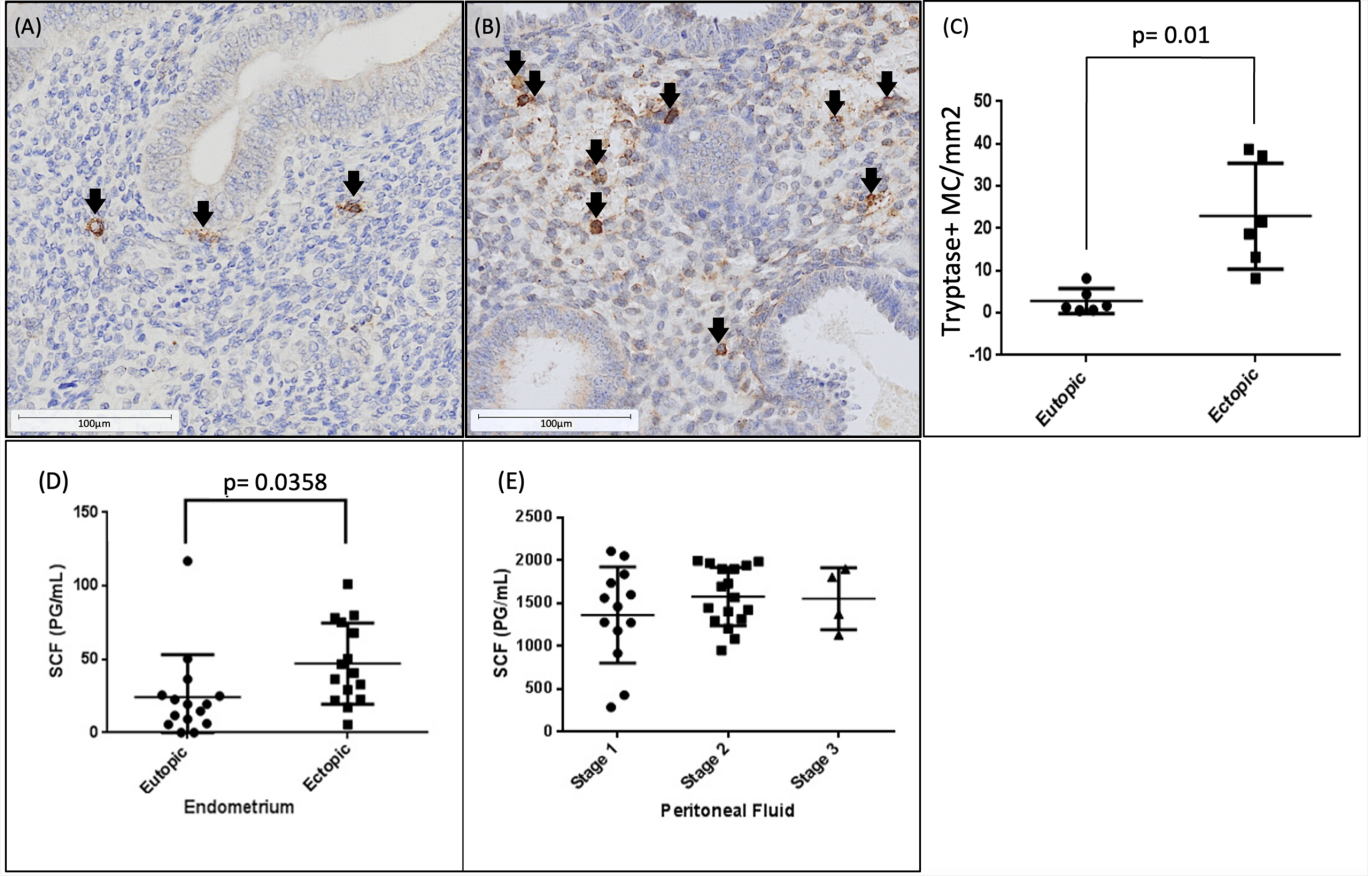
Significantly higher mast cell numbers and stem cell factor concentrations found in ectopic endometriotic tissue than eutopic endometrium. Activated Mast cells are increased in human endometriotic lesions compared to matched eutopic endometrium. Immunohistochemistry for mast cell tryptase in eutopic endometrial samples **(A)** and endometriotic lesions **(B)** from stage III-IV endometriosis patients (n=6). Quantitative analysis **(C)** revealed significantly higher number of mast cells/mm^2^ in ectopic lesions compared to eutopic endometrium (p<0.01). Mast cell tryptase positive cells are indicated by black arrowheads. Scale bars: **(A)** 150µm, **(B)** 300µm, **(C)** 75µm, **(D)** 100µm. Stem cell factor concentration in human endometriotic lesions vs eutopic endometrium (n=15) **(D)**, SCF concentrations in peritoneal fluid of endometriosis patients (n=14) stratified by disease stage **(E)**.

Mast cell tryptase is the most abundant secretory granule-derived serine proteinase contained in *mast cell granules* and has been established as a histological marker of *mast cell*s. To detect mast cells in endometriosis lesions, immunohistochemistry staining of mast cell tryptase was performed for stage III-IV endometriosis patient lesions and eutopic endometrium from matched patients (**Figures 2A, B**). A significantly higher mast cells density (p<0.01) was found in endometriotic lesions compared to eutopic endometrium in paired samples from the same patients (**Figure 2C**).

### Human endometriotic lesions produce higher concentration of SCF compared to matched eutopic endometrium

Since SCF is required for maturation, proliferation, and chemotaxis of mast cells in peripheral tissues, the levels of SCF were determined by ELISA in tissue lysates prepared from human endometriotic lesions, matched eutopic endometrium and peritoneal fluid samples. Significantly higher levels of SCF were observed in tissue lysate from endometriotic lesions compared with matched eutopic endometrium (p< 0.05, **Figure 2D**). To observe whether SCF levels associate with disease severity, we stratified SCF concentrations in peritoneal fluid of women with endometriosis into three clinical disease stages (Stage 1, 2, and 3 corresponding to minimal, mild, and severe). Although SCF concentration in the peritoneal fluid was comparatively higher than tissue lysates from endometriotic lesions, no significant differences in SCF levels in peritoneal fluid were found between the disease stages (**Figure 2E**).

### Mast cell secretions in response to estrogen and progesterone prime endometriotic epithelial cells and endometrial stromal cells to increase pro-inflammatory and chemokinetic signaling

**Figure 3:**
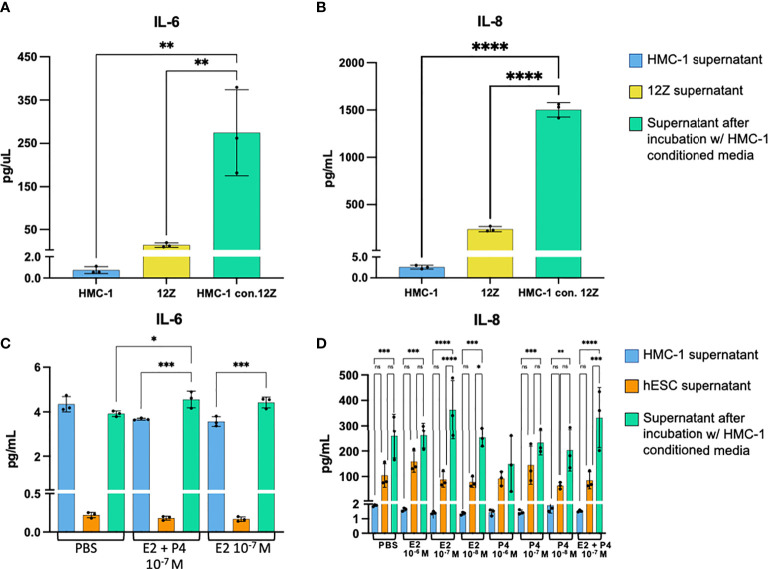
Cytokine levels in 12Z and hESC supernatant without vs. with HMC-1 conditioned media. Concentration of IL-6 **(A)** and IL-8 **(B)** in 12Z supernatant without vs. with stimulation by HMC-1 conditioned media. Concentration of IL-6 **(C)** and IL-8 **(D)** in hESC supernatant without vs. with HMC-1 conditioned media in hormonal conditions. ****p= <0.0001, ***p= 0.0001—0.001, **p= 0.001—0.008, *p< 0.05.

To understand the cross talk between MCs stimulated with estrogen and progesterone and cells of the lesion, 12Z and hESC cells were cultured with HMC-1 conditioned media primed with estrogen and progesterone. Estrogen and progesterone treatment at various concentrations (1x10^-6^ M, 1x10^-7^ M, 1x10^-8^ M, equal mixture 1x10^-7^ E2 and P4, PBS as control) did not significantly influence production of pro-inflammatory cytokines by 12Z or hESC cells compared to media controls. However, in response to HMC-1 conditioned media, 12Z cells produced significantly higher levels of IL-6 and IL-8 (p=0.0001–0.04; **Figures 3A, B**) compared to unconditioned media. Quite a similar effect was observed in hESC cultures; hESC cells increased IL-6 production when exposed to HMC-1 conditioned media specifically with a medium dose of E2 (10^-7^ M) or combination E2 + P4 (10^-7^ M) (**Figure 3C**). As well, hESC cells increased production of IL-8 in response to HMC-1 conditioned media, especially in medium and low doses of E2 (10^-7^, 10^-8^ M) and combination E2 + P4 (**Figure 3D**). IL-6 and IL-8 are both pro-inflammatory cytokines, and IL-6 is important in proliferation and maturation of MCs (20) while IL-8 acts to recruit other immune cells such as neutrophils (21).

### Estrogen increases pro-inflammatory, chemokinetic, angiogenic cytokine levels in plasma

**Figure 4:**
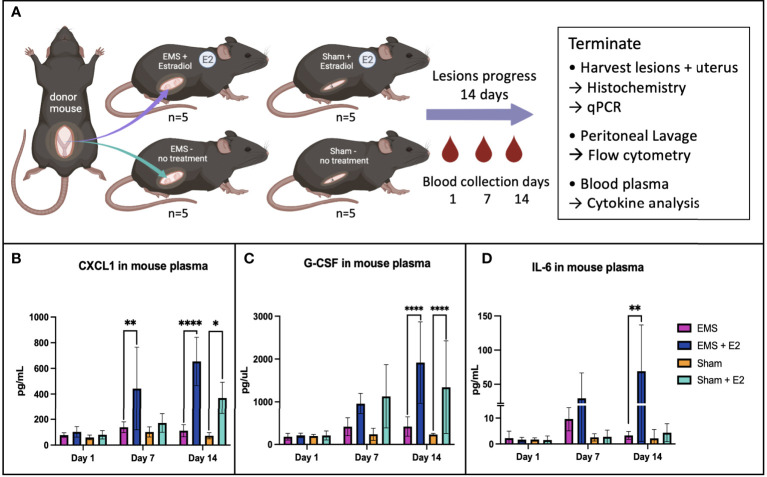
Schematic diagram of syngeneic mouse model experiment used in this study **(A)**. Plasma cytokine levels of CXCL1 **(B)**, G-CSF **(C)** and IL-6 **(D)** were found to increase significantly in the estrogen treated EMS-induced group. *p<0.05, **p<0.0055, ****p<0.0001.

To evaluate the influence of estrogen on major pro-inflammatory cytokines relevant to endometriosis pathophysiology and mast cell recruitment, we induced endometriosis in C57Bl6 immunocompetent mice supplemented with or without estrogen. Plasma cytokine levels were measured at days 1, 7 and 14 post-induction of endometriosis (**Figure 4A**). The plasma concentrations of CXCL1 and G-CSF on day 14 were significantly increased in both the E2-treated EMS-induced group as well as E2-treated sham group as compared to their non-treated counterparts (**Figures 4B, C**). The plasma concentration of IL-6 was significantly increased in the E2-treated, EMS-induced group of mice on day 14 (p=0.0015, **Figure 4D**) compared to E2-treated sham, non-treated EMS-induced, or non-treated sham groups.

### Peritoneal mast cell progenitors increase in a mouse model of endometriosis, mature mast cells decrease

**Figure 5:**
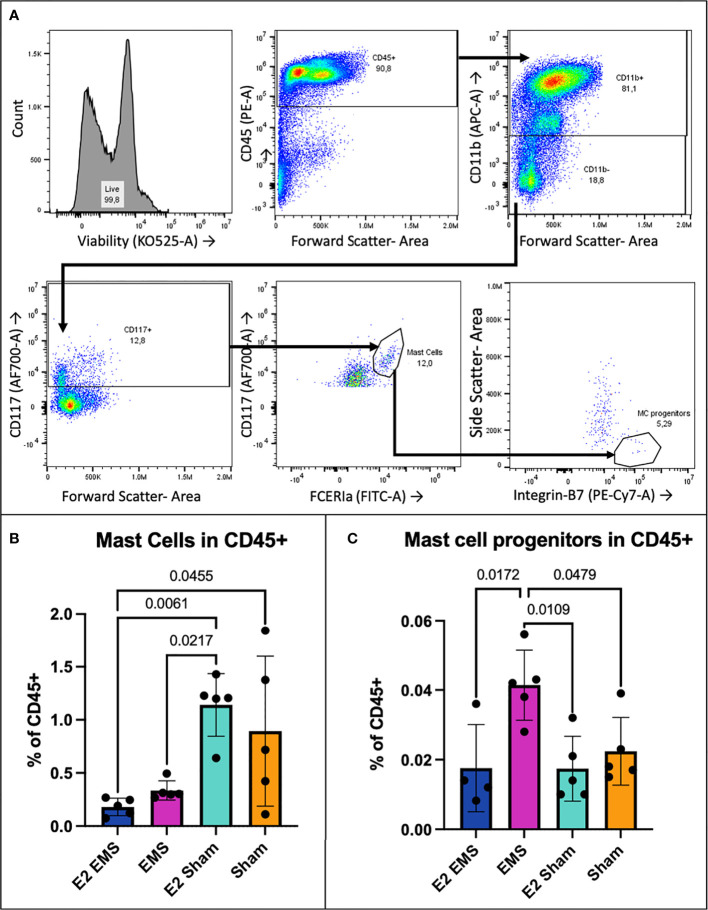
Mast cells in peritoneal fluid from mice induced with endometriosis. Mature mast cells were CD117^+^, FCERIa^+^, CD11b^-^ **(A)**. MCp were CD117^+^, FCERIa^+^, CD11b^-^, integrin β7^+^, SSC^lo^ **(A)**. The estrogen treated sham operated group had significantly higher mast cell frequency within the CD45+ population compared to the untreated EMS group (p= 0.0256) **(B)**. The E2 EMS group had significantly lower peritoneal mast cells than both E2 sham and untreated sham groups (p= 0.0057, 0.0414) **(B)**. Frequency of MCp in the CD45+ population was significantly higher in EMS-induced mice vs. E2-treated EMS and E2-sham, untreated sham groups (p= 0.0172, 0.0109, 0.0479) **(C)**.

To distinguish whether mast cells are recruited at the lesion site or if they were already present, the frequency of mast cell progenitors (MCp) were compared to mature mast cells (MC) in the PE—CD45+ population of mouse peritoneal cells. Mature MC were identified by Alexa700—CD117 (SCF receptor) and FITC—FCERIα antibody markers (**Figure 5A**), while MCp were identified by Alexa700—CD117, FITC—FCERIα and PE/Cy-7—integrin-β7 antibody markers as well as low side-scatter (**Figure 5A**). Cells positive for APC—CD11b were excluded as mast cells do not express CD11b. MCp populations were subtracted from MC populations to identify mature MC population frequency. Flow cytometry analysis showed that, while there was no significant difference of mature MCs between the EMS and E2-EMS groups, the E2-Sham group showed significantly higher frequency of MCs within the CD45+ population compared to both E2-EMS and EMS groups (p= 0,0061; 0.0217, **Figure 5B**). The frequency of MCp (CD117+ FCERIa^+^ integrin-β7+ CD11b^–^ cells) within the CD45^+^ population was considerably higher in the EMS-induced group of mice compared to E2-treated EMS-induced, E2-treated sham or untreated sham groups (p= 0.0172, 0.0109, 0.0479; **Figure 5C**). Given the overlapping functions and lineage between murine MCs and basophils, we teased out presence of peritoneal basophils in the same mice. We evaluated basophil populations in mouse peritoneal cells by identifying them as FCERIa^+^ CD117^-^ Integrin-β7^-^ CD45^int^ (**Supplemental Figure 2A**). The E2 + EMS group had basophil populations significantly smaller than both the E2 sham and untreated sham groups, expressed as frequency within the live population (p= 0.0252, 0.0141) (**Supplemental Figure 2B**).

### Estrogen upregulates gene expression of several mast cell-related genes in mouse endometriotic lesion tissue

**Figure 6:**
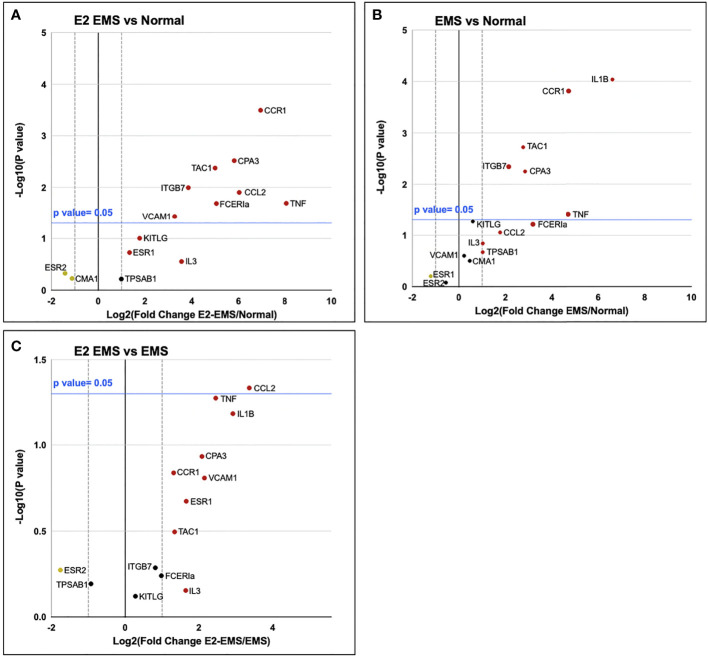
Mast cell relevant genes are upregulated in endometriotic lesion tissue. Gene transcript fold change in estrogen-treated endometriotic lesion (n=5) vs normal healthy endometrium (n=5) **(A)**, untreated endometriotic lesion (n=5) vs normal healthy endometrium **(B)**, and estrogen-treated endometriotic lesion vs untreated endometriotic lesion **(C)** of endometriosis-induced and healthy control mice. Vertical dotted lines represent a fold change of ±2, where data points outside this range have shown a fold change of more than 2.0. The blue horizontal line denotes p= 0.05 in -Log10, where data points above the blue line are significantly upregulated in the tested group.

A panel of target genes (**Table 2**) similar to the panel investigated in human tissues was applied in qPCR analysis of harvested mouse lesions in order to discern the impact of estrogen on the expression of these genes in endometriotic-like tissues. Comparing estrogen-treated lesions to control endometrium, those significantly upregulated (p ≤ 0.05) include: FCERIa, CCL2, CPA3, VCAM1, ITGB7, TNF, TAC1, CCR1, and IL1B (**Figure 6A**). In untreated lesions vs control endometrium, significantly upregulated genes include: TNF, CPA3, ITGB7, TAC1, CCR1, and IL1B, in addition FCERIa was upregulated with p= 0.06 (**Figure 6B**). CCL2 was significantly upregulated in estrogen-treated lesions compared to untreated lesions (**Figure 6C**).

**Table 2:** Genes targeted in PCR array for mouse lesion tissue expression of MC-relevant transcripts.

Gene	Protein encoded	Function in MC biology	E2-EMS vs. normal	P value	EMS vs. normal	P value	E2-EMS vs. EMS	P value
IL1B	Interleukin 1-β	Recruitment	*+*	*0.0001*	*+*	*9.2E-5*	+	0.065
IL3	Interleukin 3	Recruitment, differentiation	+	0.279	+	0.144	+	0.702
VCAM1	Vascular adhesion protein 1	Recruitment	*+*	*0.037*	/	0.251	+	0.155
CCL2	C-C Motif chemokine ligand 2	Recruitment	*+*	*0.013*	+	0.088	*+*	*0.046*
TAC1	Preprotachykinin-1 AKA “Substance P”	Differentiation	*+*	*0.004*	*+*	*0.002*	+	0.320
CMA1	Mast cell chymase 1	Protease (marker of differentiation)	–	0.591	/	0.314	–	0.080
TPSAB1	Mast cell tryptase	(marker of differentiation)	/	0.607	+	0.214	/	0.641
TNF	Tumor necrosis factor	Activation/function	*+*	*0.02*	*+*	0.039	+	0.053
KITLG	Stem cell factor (kit ligand; c-kit)	Differentiation, recruitment	+	0.099	/	0.054	/	0.758
CCR1	C-C Motif chemokine receptor 1	Activation/function	*+*	*0.0003*	*+*	*1.5E-4*	+	0.145
CPA3	Mast cell carboxypeptidase 3	Function (connective tissue MCs only)	*+*	*0.003*	*+*	*0.006*	+	0.116
FCERIa	Fc Epsilon receptor 1α	Activation	*+*	*0.02*	+	0.061	/	0.576
ITGB7	Integrin β-7	Differentiation	*+*	*0.01*	*+*	*0.005*	/	0.517

“**+**”, significantly upregulated (fold change > 1.0; p<0.05), “+”, upregulated (fold change higher than 1.0), “-”, downregulated (fold change lower than -1.0), “/”, no observed difference (-1.0 < fold change < 1.0).

### Estrogen increases mast cell numbers within mouse endometriotic lesion

**Figure 7:**
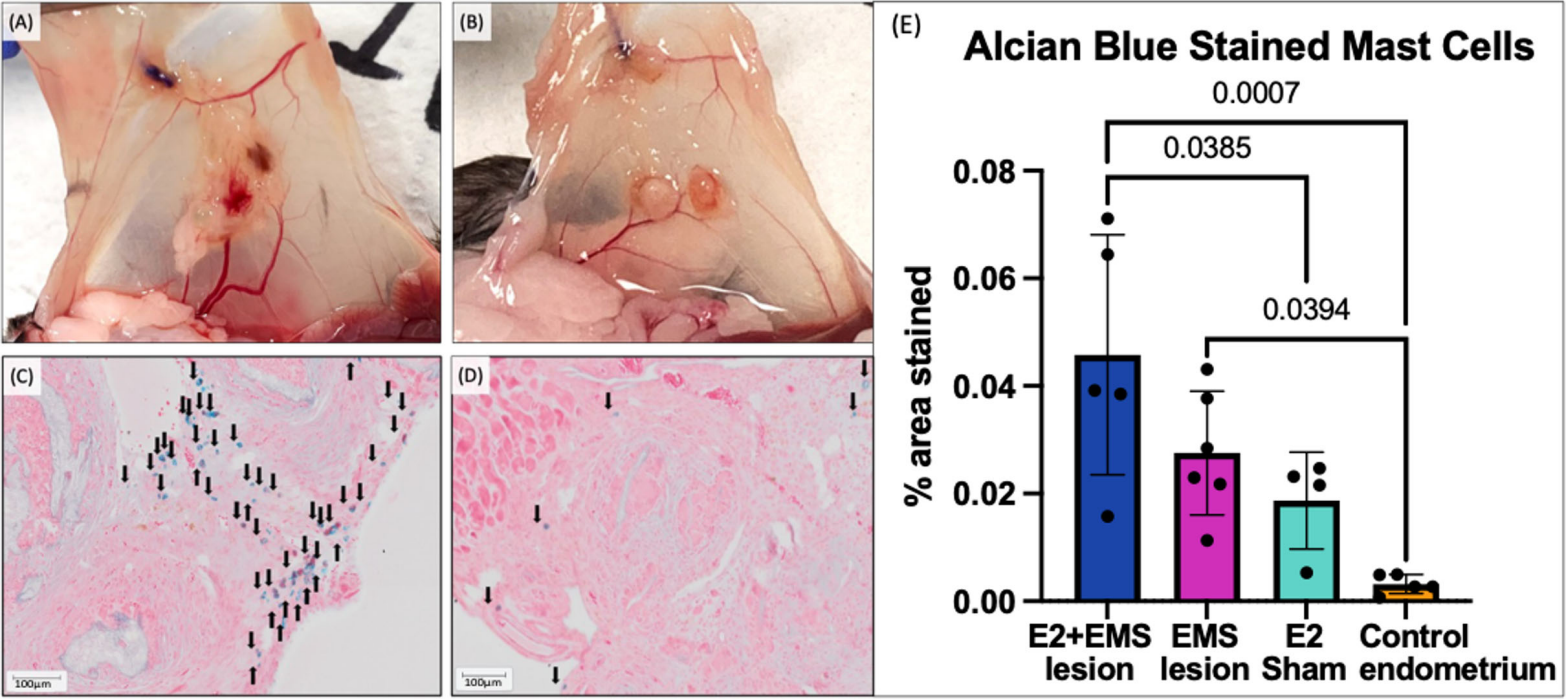
Murine endometriotic lesions, histochemistry, comparison of lesion MC presence to original uterine graft tissue. Visually notable qualitative size difference in estrogen-treated **(A)** vs untreated **(B)** mouse endometriotic lesions. Alcian blue staining of estrogen treated **(C)** and untreated **(D)** mouse endometriotic lesions, mast cells are denoted with black arrows. The % area stained with Alcian blue in lesions from E2-EMS mice (n= 5) and untreated EMS mice (n=6) was significantly higher compared to control endometrium (n= 5) (p= 0.0007, 0.0394) **(E)**. Alcian blue staining was also significantly higher in E2-EMS lesions compared to endometrium of E2-sham mice (n=4) (p=0.0385). Scale bars: 100 μm **(C, D)**.

To evaluate the difference of mast cell presence in endometrial tissue before engrafting as endometriotic lesions, the harvested lesions were compared against segments preserved from donor mouse uterus. The presence of mast cells in endometriotic lesions and control endometrium obtained from C57BL/6 mice (**Figures 7A, B**) was determined using Alcian blue histochemical stain. Alcian blue stained mast cells were detected within both ectopic lesions and control endometrial tissue of these mice (**Figures 7C, D**). Alcian blue stained MCs were significantly higher in lesions obtained from E2-treated mice vs. control endometrium (p= 0.0011), and to a lesser extent, MCs were significantly higher in untreated lesions than control endometrium (p= 0.0367) (**Figure 7E**). To address impact of estrogen on proliferation and angiogenesis of mouse endometriotic lesions, immunohistochemical staining was conducted to examine Ki67 and CD31 antibody markers (**Supplemental Figure 1**). Our analysis did not identify significant differences between groups for either stain (**Supplemental Figures 1F, G**).

## Discussion

The etiological mechanisms of endometriosis remain poorly defined yet immune-endocrine pathways are central to the pathogenesis. Given the important role of inflammation in the disease, further understanding of the influence of specific immune cell populations is critical. Mast cells are of particular interest due to previously noted correlations between occurrences of endometriosis and allergic diseases. In addition, mast cells release factors involved in inflammation, pain, fibroblast proliferation, and angiogenesis—processes known to be involved in the pathogenesis of endometriosis (22). It is not yet determined whether mast cells contribute to the progression of endometriosis or if they are drawn to the lesion in response to existing inflammation, and what role they play in the pathophysiology of the disease.

The differences seen in transcript levels for genes relevant to mast cell biology in human patient samples (**Figure 1**) illustrate a distinction between the endometriotic lesion and normal endometrium in terms of a microenvironment conducive to mast cell recruitment. For example, the upregulation of KITLG (SCF) in endometriotic lesion tissue is especially telling of a microenvironment which recruits mast cells and supports their maturation and activation. Vascular cell adhesion protein 1 (VCAM-1) and C-C Motif chemokine ligand 2 (CCL2) are both important in recruiting mast cells to inflamed tissues, and CCL2 is known to activate mast cells (23, 24). The increased transcript levels for genes of mast cell activation proteins like mast cell carboxypeptidase 3 (CPA3) and mast cell chymase (CMA1) point to active mast cell differentiation, since the formation of these proteins is rapidly upregulated as during mast cell maturation. Mast cell tryptase (TPSAB1) is a reliable MC marker as tryptase is the protease common to all MC phenotypes and indicates MC maturation. Chymase 1 (CMA1) is a significant marker of mast cell differentiation and function, as chymase is required to degrade the extracellular matrix when mast cells are degranulating, meaning an increase in CMA1 transcripts would indicate new formation of mature mast cells equipped to be activated (25). Mast cell carboxypeptidase A (CPA3) is an essential mast cell protease, and an increased CPA3 transcript level has been acknowledged as a reliable biomarker for allergic inflammation (26). The higher TNF transcript levels coinciding with mast cell growth factor transcripts suggests an increased production of TNF-α by mast cells, since mast cells secrete TNF-α for their roles in proliferation and angiogenesis (27). An upregulation of C-C Motif chemokine receptor 1 (CCR1) is noteworthy for a few reasons. Firstly, its ligand (CCL8) has been identified as a proinflammatory signalling molecule and chemokine for mast cells (28). More interestingly, a crosstalk relationship has recently been described by Li *et al. (* 28) wherein mast cells, *via* the CCL8/CCR1 axis, were found to promote proliferation of endometriotic epithelial cells. As well, mast cells increased migration of endometrial stromal and vascular endothelial cells in response to CCL8 (28). These effects were seen in our cell co-culture and a mouse model of endometriosis, and are worth consideration in the present study and future investigations (28).

Mast cell tryptase is a specific marker for mature mast cells (29). Immunohistochemical staining with anti-human mast cell tryptase antibody revealed a significantly higher number of mast cells in human endometriotic lesions compared with matched eutopic endometrium, while ELISA with anti-human SCF revealed increased SCF concentrations in the same. This finding is supportive of previous reports of increased mast cell numbers in endometriosis (5). Higher numbers of tryptase positive mast cells in endometriosis lesions potentially suggest that activated mast cells infiltrate these lesions during the progression of the disease or that mast cells are already present prior to lesion formation and contribute to the initial lesion development. Regardless of their presence during initiation or establishment of endometriosis lesion development, their increased numbers suggest that mast cells likely contribute to localized peritoneal inflammation.

In the current study, significantly higher concentration of SCF was found in endometriotic lesions compared to the matched eutopic endometrium obtained from same patients. These results are supportive of previous studies that have measured the concentration of SCF in peritoneal fluid of women with endometriosis compared to non-endometriosis controls (13). The significantly higher SCF concentration paired with a significantly higher number of mast cells suggests heightened mast cell chemotaxis and infiltration in endometriosis lesions. Indeed, our findings confirm that human endometriotic lesions harbour an ideal microenvironment to induce differentiation, maturation, and secretory function of mast cells by increasing the concentration of SCF.

Our *in vitro* experiments revealed interesting crosstalk between mast cells, endometriotic epithelial cells and endometrial stromal cells. The increase in IL-6 and IL-8 production by endometriotic epithelial 12Z cells after exposure to HMC-1 secretions indicates a communication by mast cells to prompt endometriotic epithelial cells to promote inflammation, mast cell proliferation and reactivity (20), neutrophil recruitment (21), and angiogenesis (30). In hESC cells, similar effects were seen only in medium or low doses of estrogen or estrogen + progesterone combination. The recruitment of neutrophils by MCs is well documented (31, 32), though the HMC-1 cell line has been documented to express very little tryptase (33). The exact signalling pathway that induced the observed response 12Z and hESC cells is worth further investigation as crosstalk between mast cells and epithelial or stromal endometrial cells concerning neutrophil recruitment have yet to be clearly elucidated.

In order to gain insights into the impact of estrogen on mast cell recruitment and differentiation, we used our established immunocompetent syngeneic mouse model of endometriosis. Mice treated with 17-β-estradiol showed elevated plasma levels of IL-6, CXCL-1, and G-CSF, demonstrating a systemic-level increase in inflammation and innate immune cell activation. CXCL-1 has been documented to act in angiogenic roles alongside its traditional neutrophil recruitment role (34, 35). Elevated plasma levels of IL-6 and G-CSF have both been reported in endometriosis patients (36, 37). Flow cytometry analysis of peritoneal immune cells found mast cell progenitors are significantly increased in EMS-induced mice, while mature mast cells appear to decrease in EMS compared to sham operated mice. Low detection of mast cells from peritoneal lavage is likely owing to the tissue-resident nature of mast cells, with many residing in connective tissue. Future studies are required to more clearly define the migration of mast cells in the peritoneal microenvironment in response to tissue injury or presence of endometriotic lesion. In the lesions endometriosis-induced mice, treatment with estrogen significantly upregulated a host of genes relevant in mast cell biology. Though direct comparison of the E2-EMS group with untreated EMS group shows only CCL2 to be significantly upregulated (**Figure 6C**), looking at E2-EMS vs. Normal (**Figure 6A**) alongside EMS vs. Normal (**Figure 6B**) it appears the expression of these genes occur differently with estrogen treatment. In histochemical analysis of mouse tissues, estrogen treatment in EMS-induced mice showed significantly increased Alcian blue staining in the lesion indicating high mast cell presence compared to estrogen-treated endometrium or control endometrium. Indeed, endometriotic-like lesions have significantly more mast cells than control mouse endometrium, and the addition of estrogen boosts this presence further. This data supports a role for estrogen in modulating the lesion immune microenvironment to increase recruitment and/or activation of mast cells in the lesion.

While our study revealed mast cell conducive endometriosis lesion microenvironment, increased numbers of mast cells and SCF, a fundamental question that needs to be addressed is whether mast cells are recruited by the lesion microenvironment or if they are simply present as bystanders. Our syngeneic mouse model experiments did indicate that high-estrogen endometriotic lesions harbour an ideal microenvironment for mast cell recruitment. Our data show promising evidence that mast cells, under influence of estrogen, are recruited to the endometriotic lesion microenvironment and have an active role in endometriosis pathophysiology. However, more refined mouse models such as a c-kit deficient mouse model (38) or a directed mast cell ablation Cre-Lox mediated excision (39) could be useful models for observing mast-cell specific effects in the pathogenesis of endometriosis. This work stands to open new avenues of research in developing mast cell-targeted therapies that could offer relief to endometriosis patients.

## Data availability statement

The raw data supporting the conclusions of this article will be made available by the authors, without undue reservation.

## Ethics statement

The studies involving human participants were reviewed and approved by Queen’s University Health Sciences Research Ethics Board. The patients/participants provided their written informed consent to participate in this study. The animal study was reviewed and approved by University Animal Care Committee, Queen’s University, Kingston, Ontario, Canada.

## Author contributions

AM and YN conceived experiments, conducted experiments, analyzed data, and wrote the manuscript. HL, JM, KK, SA, SM, MB, and DS helped conduct experiments. AC contributed reagents. SY and BL contributed human patient samples. MK and CT contributed reagents, conceived experiments, provided financial support, and editing of manuscript. All authors contributed to the article and approved the submitted version.

## Funding

This research was supported with funds from Canadian Institutes of Health Research (CIHR 394924, CT and MK)

## Conflict of interest

The authors declare that the research was conducted in the absence of any commercial or financial relationships that could be construed as a potential conflict of interest.

## Publisher’s note

All claims expressed in this article are solely those of the authors and do not necessarily represent those of their affiliated organizations, or those of the publisher, the editors and the reviewers. Any product that may be evaluated in this article, or claim that may be made by its manufacturer, is not guaranteed or endorsed by the publisher.

## Supplementary material

Supplementary Figure 1 Immunohistochemical staining of CD31, Ki67 in endometriotic lesions. Immunohistochemical staining of CD31 in estradiol treated **(A)**, non-treated **(B)** murine endometriotic lesions, and donor control endometrium **(C)**. Ki67 staining in estradiol treated **(D)**, non-treated **(E)** murine endometriotic lesions and control endometrium **(F)**. No statistically significant differences were observed between groups for either stain **(G, H)**. Scale bars: 100 μm.

Supplementary Figure 2 Basophil populations in mouse peritoneal fluid. Basophils were identified as FCERIa^+^ CD117^-^ integrin-B7^-^ with intermediate CD45 expression **(A)**. Basophil populations were significantly lower in the E2 + EMS group compared to E2 sham (p= 0.0252) and untreated sham (p=0.0141) **(B)**.

## Abbreviations

EMS, Endometriosis; IL, Interleukin; MC, Mast cell; MCcp, Committed mast cell progenitor; MCT, Mast cell tryptase; SCF, Stem cell factor.
