# Supplementary figures and images for: Estrogen mediates inflammatory role of mast cells in endometriosis pathophysiology
Source: Front Immunol. 2022 Aug 9;13:961599. doi: 10.3389/fimmu.2022.961599 (PMC9396281; doi:10.3389/fimmu.2022.961599)

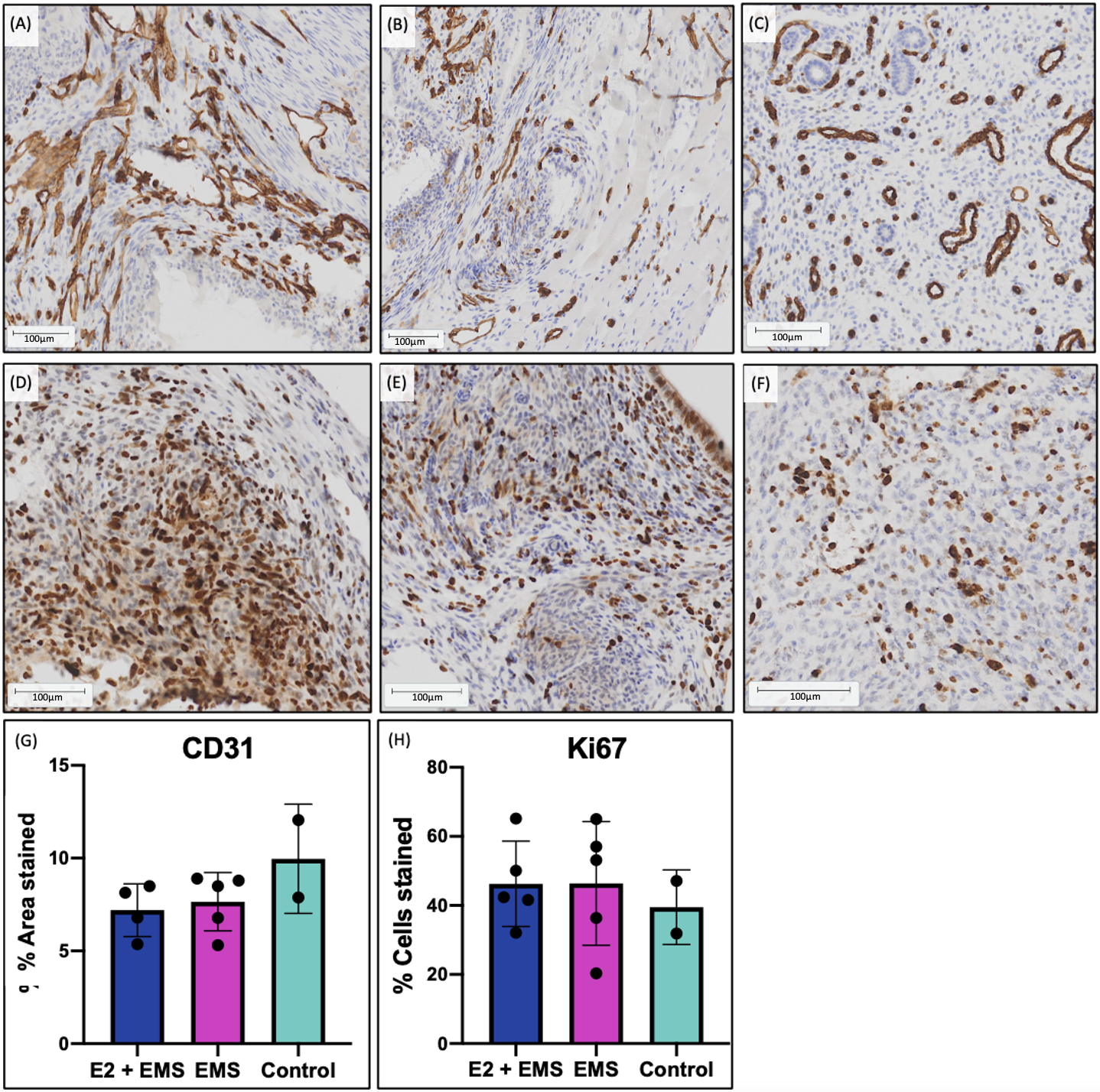

Supplement: Supplementary Figure 1 — Immunohistochemical staining of CD31, Ki67 in endometriotic lesions. Immunohistochemical staining of CD31 in estradiol treated (A), non-treated (B) murine endometriotic lesions, and donor control endometrium (C). Ki67 staining in estradiol treated (D), non-treated (E) murine endometriotic lesions and control endometrium (F). No statistically significant differences were observed between groups for either stain (G, H). Scale bars: 100 μm. [file Image_1.tiff]

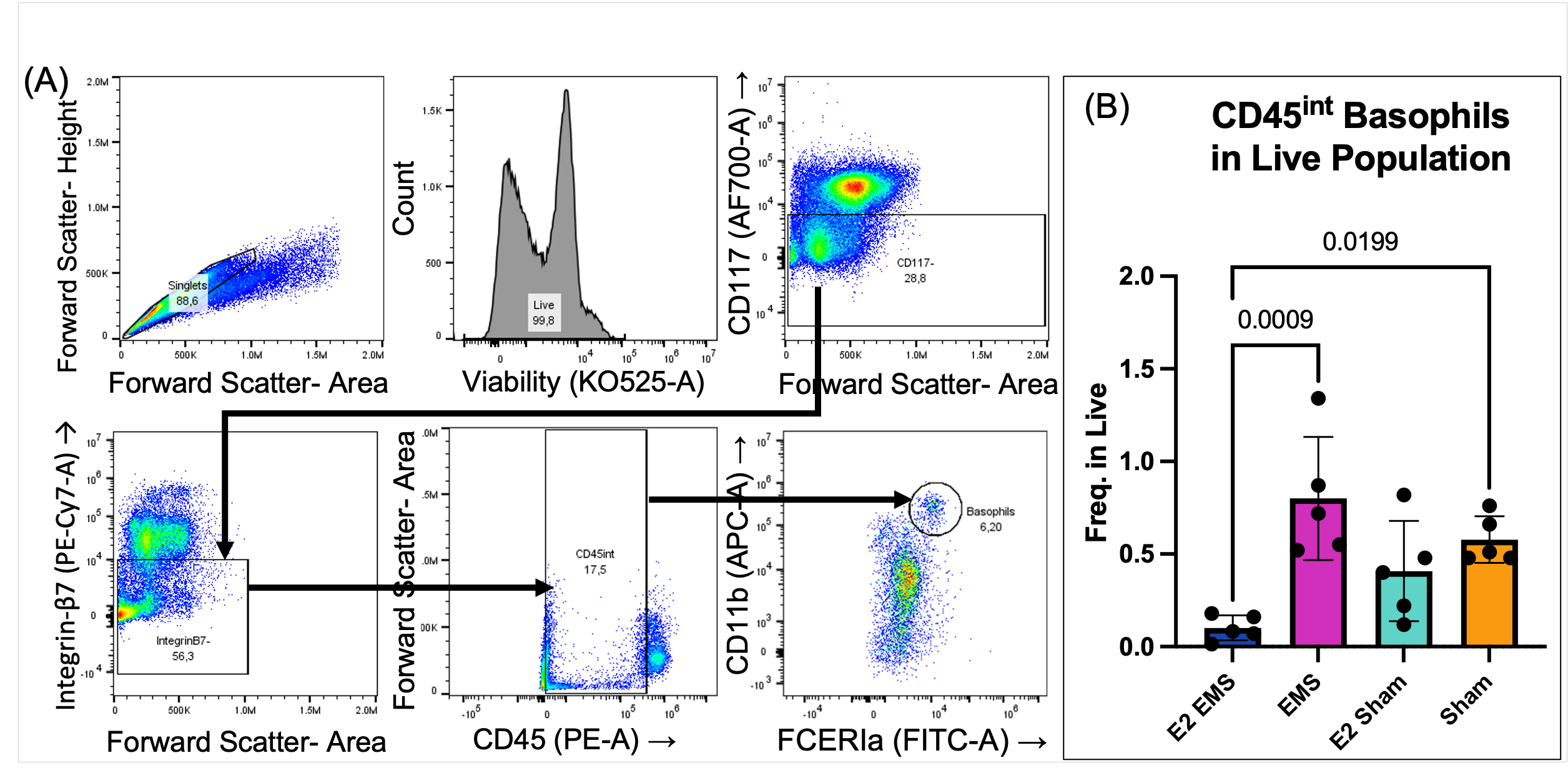

Supplement: Supplementary Figure 2 — Basophil populations in mouse peritoneal fluid. Basophils were identified as FCERIa+ CD117- integrin-B7- with intermediate CD45 expression (A). Basophil populations were significantly lower in the E2 + EMS group compared to E2 sham (p= 0.0252) and untreated sham (p=0.0141) (B). [file Image_2.tiff]
